# Safety and efficacy of Cerebrolysin in motor function recovery after stroke: a meta-analysis of the CARS trials

**DOI:** 10.1007/s10072-017-3037-z

**Published:** 2017-07-13

**Authors:** Alla Guekht, Johannes Vester, Wolf-Dieter Heiss, Eugene Gusev, Volker Hoemberg, Volker W. Rahlfs, Ovidiu Bajenaru, Bogdan O. Popescu, Edith Doppler, Stefan Winter, Herbert Moessler, Dafin Muresanu

**Affiliations:** 1 Moscow Research and Clinical Center for Neuropsychiatry and Russian National Research Medical University, Moscow, Russia; 2 Department of Biometry and Clinical Research, IDV Data Analysis and Study Planning, Krailling, Germany; 3 Max Planck Institute for Metabolism Research, Cologne, Germany; 4 Department of Neurology, Neurosurgery and Medical Genetics, Pirogov Russian National Research Medical University, Moscow, Russia; 5 Department of Neurology, SHR Gesundheitszentrum Bad Wimpfen GmbH, Bad Wimpfen, Germany; 6 Department of Neurology, “Carol Davila” University of Medicine and Pharmacy, Bucharest, Romania; 7 Laboratory of Molecular Biology, ‘Victor Babes’ National Institute of Pathology, Bucharest, Romania; 8 Department of Clinical Research, EVER Neuro Pharma GmbH, Unterach, Austria; 9 COMAMO LifeSciences GmbH, Mondsee, Austria; 10 Department of Clinical Neurosciences, “Iuliu Hatieganu” University of Medicine and Pharmacy, Victor Babes Street No. 8, 400012 Cluj-Napoca, Romania; 11 “RoNeuro” Institute for Neurological Research and Diagnostic, Strada Mircea Eliade 37, 400000 Cluj-Napoca, Romania

**Keywords:** Cerebrolysin, Stroke, Recovery, Early benefit, NIHSS, Meta-analysis

## Abstract

This meta-analysis combines the results of two identical stroke studies **(**CARS-1 and CARS-2) assessing efficacy of Cerebrolysin on motor recovery during early rehabilitation. Cerebrolysin is a parenterally administered neuropeptide preparation approved for the treatment of stroke. Both studies had a prospective, randomized, double-blind, placebo-controlled design. Treatment with 30 ml Cerebrolysin once daily for 3 weeks was started 24–72 h after stroke onset. In addition, patients participated in a standardized rehabilitation program for 21 days that was initiated within 72 h after stroke onset. For both studies, the original analysis data were used for meta-analysis (individual patient data analysis). The combination of these two studies by meta-analytic procedures was pre-planned, and the methods were pre-defined under blinded conditions. The nonparametric Mann-Whitney (MW) effect size of the two studies on the ARAT score on day 90 indicated superiority of Cerebrolysin compared with placebo (MW 0.62, *P* < 0.0001, Wei-Lachin pooling procedure, day 90, last observation carried forward; *N* = 442). Also, analysis of early benefit at day 14 and day 21 by means of the National Institutes of Health Stroke Scale, which is regarded as most sensitive to early improvements, showed statistical significance (MW 0.59, *P* < 0.002). The corresponding number-needed-to-treat (NNT) for clinically relevant changes in early NIHSS was 7.1 (95% CI: 4 to 22). Cerebrolysin had a beneficial effect on motor function and neurological status in early rehabilitation patients after acute ischemic stroke. Safety aspects were comparable to placebo, showing a favourable benefit/risk ratio.

## Introduction

After the failure of monomodal neuroprotective therapy to demonstrate benefit in acute ischemic stroke [1], new concepts must be developed to use multimodal therapeutic strategies, which might not only diminish tissue damage in the acute phase but additionally support neuroplasticity and thereby improve recovery. In order to achieve an effect in neurorehabilitation, the treatment must be given early and longer than the neuroprotective paradigm after stroke, which is usually up to 10 days, and these therapeutic interventions should be combined with rehabilitative measures stimulating functional abilities. This concept was the basis for two identical, randomized, placebo-controlled, double-blind trials (CARS-1 and CARS-2) evaluating the effect of a 3-week treatment with Cerebrolysin, a neuropeptide preparation with multimodal action, on stroke recovery [2, 3]. The present meta-analysis of both studies was pre-planned under blinded conditions, with the nonparametric methodology operationalized in the CARS-2 final statistical analysis plan.

## Methods

### Identification of studies and collection of data

This is a formal meta-analysis of two stroke studies on Cerebrolysin of identical design: the CARS-1 study, which was performed in the EU and the Ukraine [4] and the CARS-2 study, which was performed in Russia. For both studies, the original analysis data were used for the meta-analysis (individual patient data [IPD] analysis), thus ensuring a high degree of methodological consistency ﻿[5]﻿. Risk of bias is provided in Table 1.

**Table 1:** Assessment of risk of bias: quality measures of the included trials

Trials	Concealment of randomization	RCT stopped early	Patients blinded	Health care providers blinded	Data collectors blinded	Outcome assessors blinded
CARS-1	Yes	No	Yes	Yes	Yes	Yes
CARS-2	Yes	No	Yes	Yes	Yes	Yes

### Statistical analyses

The pre-planned nonparametric procedure of this IPD meta-analysis was the well-known and robust Wilcoxon-Mann-Whitney (WMW) test [6–9] (the primary endpoint is a rating scale and distributions were expected to be skewed with outliers and floor-ceiling effects). The effect size measure associated to the WMW test is the Mann-Whitney (MW) measure of superiority, a highly robust effect size measure with minimized assumptions, representing the gold standard for full scale ordinal analysis [10–14].

The technical expression for the MW is [P(X < Y) + 0.5 P(X = Y)]. The traditional benchmarks for the MW effect size measure are as follows [15, 16]: 0.29 = large inferiority; 0.36 = medium inferiority; 0.44 = small inferiority; 0.50 = equality; 0.56 = small superiority; 0.64 = medium superiority; 0.71 = large superiority.

### Handling of efficacy criteria

#### Upper limb motor function (Action Research Arm Test)

The primary efficacy criterion in both studies was the change from baseline at day 90 in the ARAT [17] score assessing the recovery of the upper limb motor function. The ARAT score ranges from 0 (no function) to 57 (no functional limitation). A target subgroup analysis was defined for patients having an ARAT baseline score > 0 (pre-planned target subset). Sensitivity analyses were performed stratified for age, gender and quartiles of ARAT baseline score.

#### Early treatment effects (NIH Stroke Scale)

Early treatment effects (day 14 and day 21) were evaluated by means of the changes from baseline of the NIHSS [18] as a secondary analysis. The NIHSS reflects neurological impairment, the clinical domain in which early effects of acute stroke therapies are likely to be most marked [19]. Recent research showed that the NIHSS in fact is most sensitive for such early points in time [19]. Furthermore, NIHSS is less influenced by extraneous factors, improving sensitivity to acute treatment effects [19]. The NIHSS score ranges from 0 (no neurological symptoms) to 42 (very severe neurological deficits). Assessors were trained on NIHSS administration.

#### Final global disability (modified Rankin Scale)

The modified Rankin scale [20] (mRS) is a functional global outcome scale measuring the level of disability after a stroke. It is a 7-point ordinal scale with a score of 0 indicative of no residual symptoms and the worst possible score of 6, which is assigned in case of death. The analysis of this secondary endpoint was performed as supportive analysis for the primary functional endpoint (ARAT day 90), based on final changes from baseline.

### Handling of safety aspects

Safety criteria were treatment-emergent adverse events (TEAEs), treatment-emergent serious adverse events (TESAEs) and death.

### Treatment of missing values

Missing values were replaced by the last observation carried forward (LOCF) technique. Observed case (OC) analysis was performed in addition as sensitivity analysis.

### Points in time

In line with the primary study endpoints of both CARS studies, day 90 was also the primary point in time of this meta-analysis. Early benefit was assessed in addition by means of day 14 and day 21 evaluations. Study visits were conducted at 7 (visit 3; V3), 14 (V4) and 21 (V5) days after baseline and on days 42 (V6) and 90 (V7) post-stroke.

### Patient populations

All analyses of this meta-analysis were performed on a modified intention-to-treat (mITT) analysis set, which included all randomized patients who have had at least one dose of study medication and have assessments for the primary endpoint at baseline and at least one time point after the first dose of study medication (pre-planned dataset definition).

### Methods of synthesis

The pre-planned method of synthesis for the Mann-Whitney (MW) effect size measure [10–14] (see also section “Statistical analyses”) was the Wei-Lachin test of stochastic ordering (one-dimensional test) [21], a maximin-efficient robust test (MERT) [22, 23], which provides a combined MW estimate and test of overall treatment effect from an ensemble of independent studies. In combination with stochastic ordering or one-dimensional alternative, it is a powerful and robust meta-analytic procedure for combining the MW effect size across studies. The one-dimensional alternative of stochastic superiority is to be interpreted as follows: at least one trial has an underlying true beneficial effect and none have an adverse effect (no qualitative interaction). In contrast to other pooling procedures, the Wei-Lachin procedure is also appropriate in case of only very few included studies, such as in the present case of two studies of identical design. Originally developed for robust pooling of subgroup results, the Wei-Lachin approach requires minimal assumptions and has been shown to be robust also with respect to presence of heterogeneity [21]. Qualitative interaction was tested by means of the Gail-Simon test [24].

As sensitivity analysis, the classic pooling procedures based on the fixed effect model (Hedges-Olkin) [25] and the random effects model (DerSimonian-Laird) [26] were calculated in addition. Associated tests for quantitative heterogeneity were performed using standard Chi-square statistic [27] and *I* ^2^ statistic [28]. It is to note that the fixed effect model is less appropriate in case of heterogeneity, while the random effects model (DerSimonian-Laird) requires a larger number of included studies for reliable estimation of between-study variance [29–31]. Thus, results of the ‘classic’ pooling procedures should be interpreted with certain care and as second line only.

## Results

### Study population

A total of 448 patients were randomized (CARS-1: 208 patients, CARS-2: 240 patients, see Table 2). All of them received study medication (safety population *N* = 448), but only 442 patients (98.7%) had an ARAT assessment after the first dose of study medication which qualified them for the mITT analysis of the ARAT on day 90. For the OC analysis, corresponding data of 429 patients (97.1% of mITT) were available. Premature discontinuation was 3.8% (CARS-1) and 4.2% (CARS-2). The overall rate of observations missing from randomized subjects was 4.2% (CARS-1: 3.9%, CARS-2: 4.6%), which is below the recommended benchmark of 10% for class I evidence-based quality studies [32–34]. The mean age of the patients was 63.8 years, the proportion of males was 59.7%, and the overall NIHSS mean was 8.1 for the combined studies. Characteristics of the included studies and patients are described in Table 2.

**Table 2:** Study and demographic characteristics of CARS-1 and CARS-2

Trials	Trial duration	Number of infusions^a^	Randomized patients (safety set), *N*	mITT patients, *N* (%)^b^	Valid ARAT, *N* (%)^c^		Mean age^d^, years	Male^d^, %	Mean NIHSS score^e^
					LOCF	OC			
CARS-1	90 days	21	208	205 (98.6%)	205 (100%)	200 (97.6%)	64.0	63.9	9.2
CARS-2	90 days	21	240	237 (98.8%)	237 (100%)	229 (96.6%)	63.5	56.5	6.8
Combined	90 days	21	448	442 (98.7%)	442 (100%)	429 (97.1%)	63.8	59.7	8.1

^a^Patients received placebo or 30 ml/day of Cerebrolysin

^b^% referring to randomized patients

^c^% referring to mITT patients

^d^% of randomized patients

^e^% of mITT patients

*ARAT* Action Research Arm Test, *LOCF* last observation carried forward, *mITT* modified intention-to-treat, *NIHSS* National Institutes of Health Stroke Scale, *OC* observed cases

### Upper limb motor function (primary final endpoint ARAT)

**Fig. 1:**
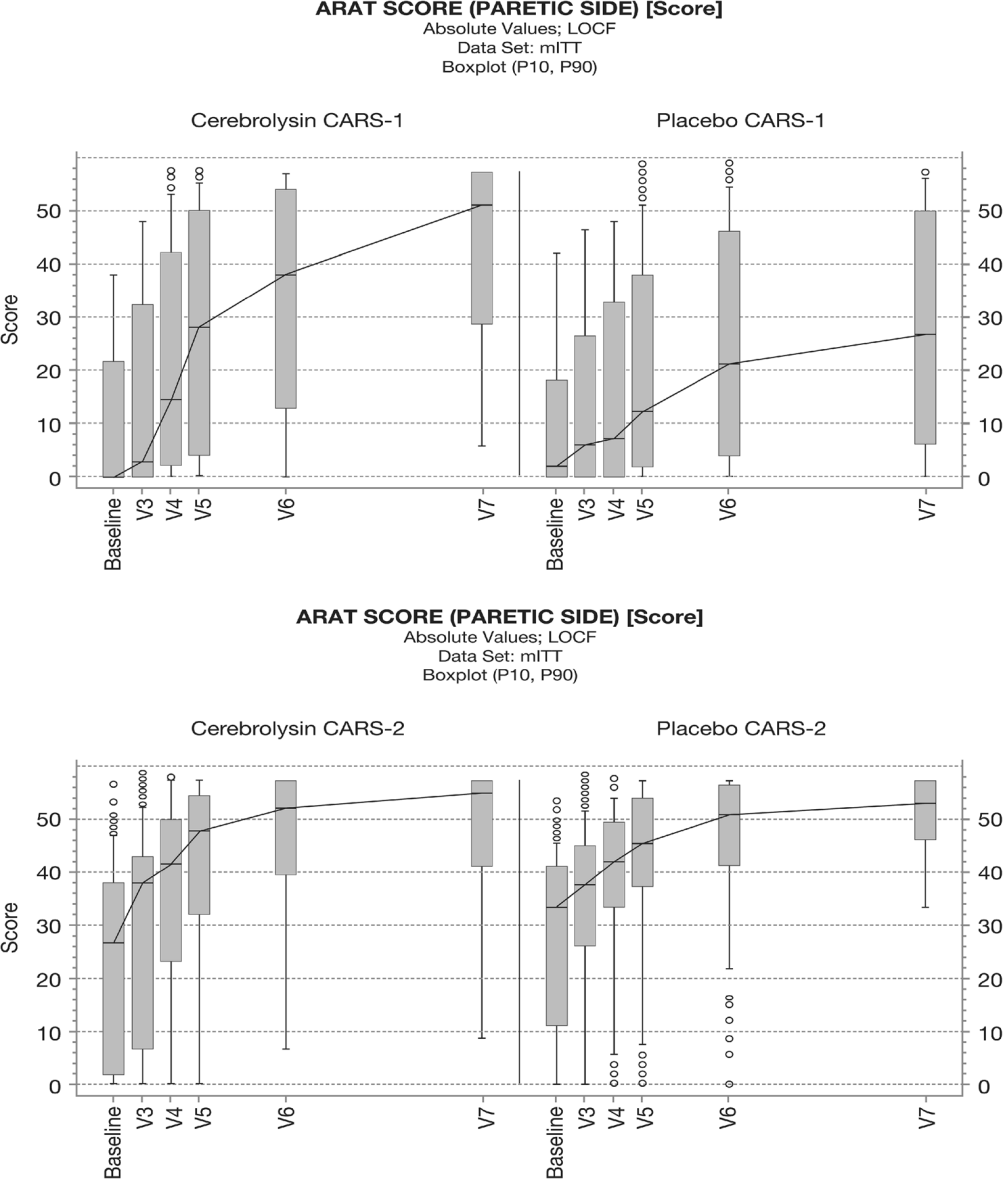
Time course of ARAT scores in the Cerebrolysin (30 ml/day) and placebo groups of CARS-1 (*upper panel*) and CARS-2 (*lower panel*). *Boxplot* (P10, P90), absolute values, mITT, LOCF

The Cerebrolysin group showed a quite similar final result at day 90 (V7) in both studies with ARAT median levels above 50, i.e., at the ceiling of the ARAT scale (final median CARS-1: 51.0, CARS-2: 55.0, Fig. 1).

**Fig. 2:**
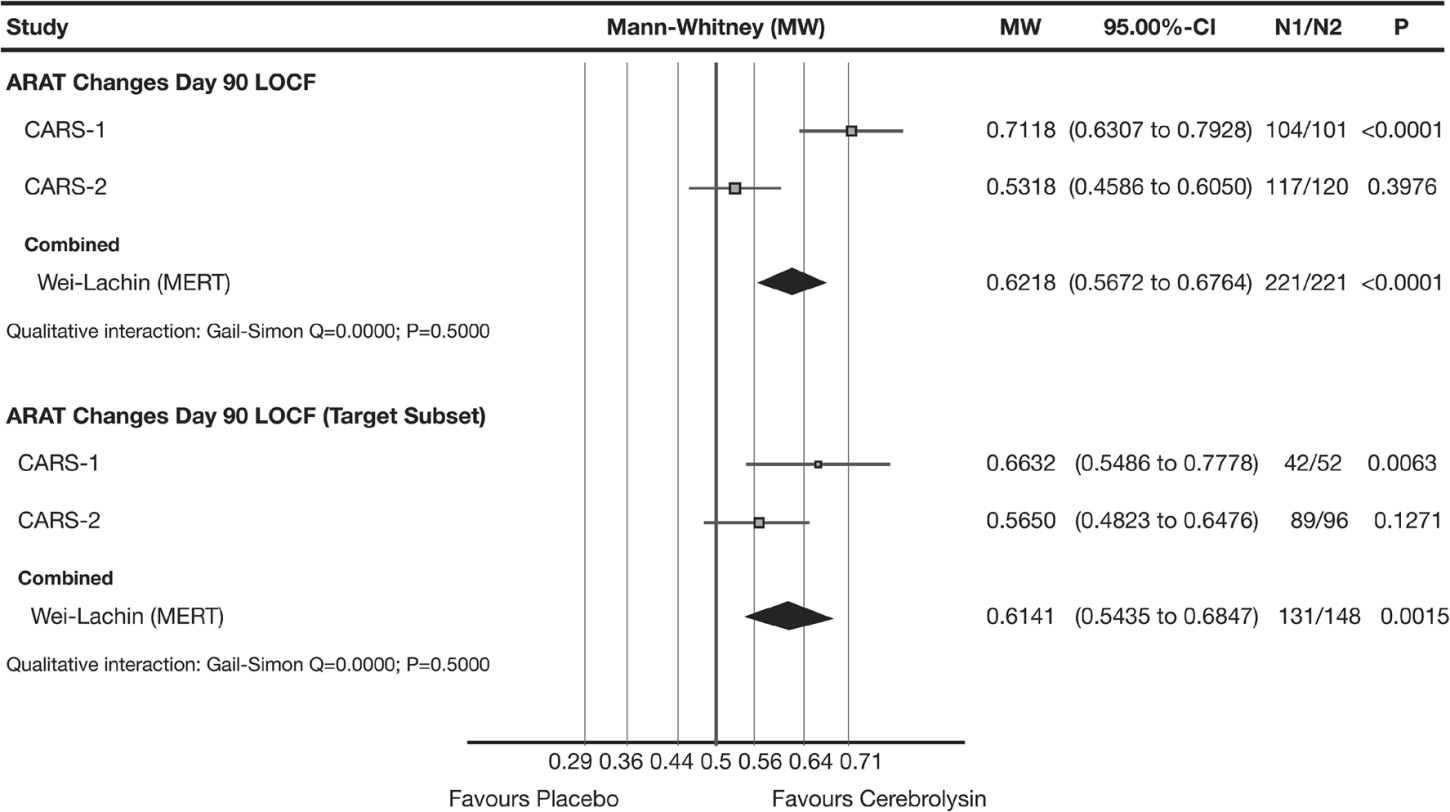
Meta-analysis of ARAT changes from baseline of CARS-1 and CARS-2. Comparison of Cerebrolysin (30 ml/day) versus placebo at day 90 in the mITT population (*upper panel*) and in the target subset with ARAT baseline >0 (*lower panel*); LOCF. Wei-Lachin pooling procedure, maximin-efficient robust test (MERT), effect size: Mann-Whitney (MW)

The a priori planned nonparametric LOCF meta-analysis showed a small to medium superiority of Cerebrolysin for the changes from baseline in the primary functional efficacy criterion ARAT at day 90, with a combined MW effect size of 0.62 (95% CI 0.57 to 0.68; *P* < 0.0001; Wei-Lachin pooling procedure [MERT], Fig. 2, upper panel).

A target subset was a priori defined in both original investigational plans excluding patients with ARAT baseline scores of ‘0ʼ. Main rationale was the fact that ARAT baseline scores of ‘0ʼ may have very different reasons, not always related to upper limb function. In the pre-planned homogeneous target subset of ARAT baseline >0, the LOCF analysis showed a small to medium superiority of Cerebrolysin with a combined MW effect size of 0.61 (95% CI 0.54 to 0.68; *P* = 0.0015; Wei-Lachin pooling procedure [MERT], Fig. 2, lower panel).

### Early treatment effects (NIHSS)

**Fig. 3:**
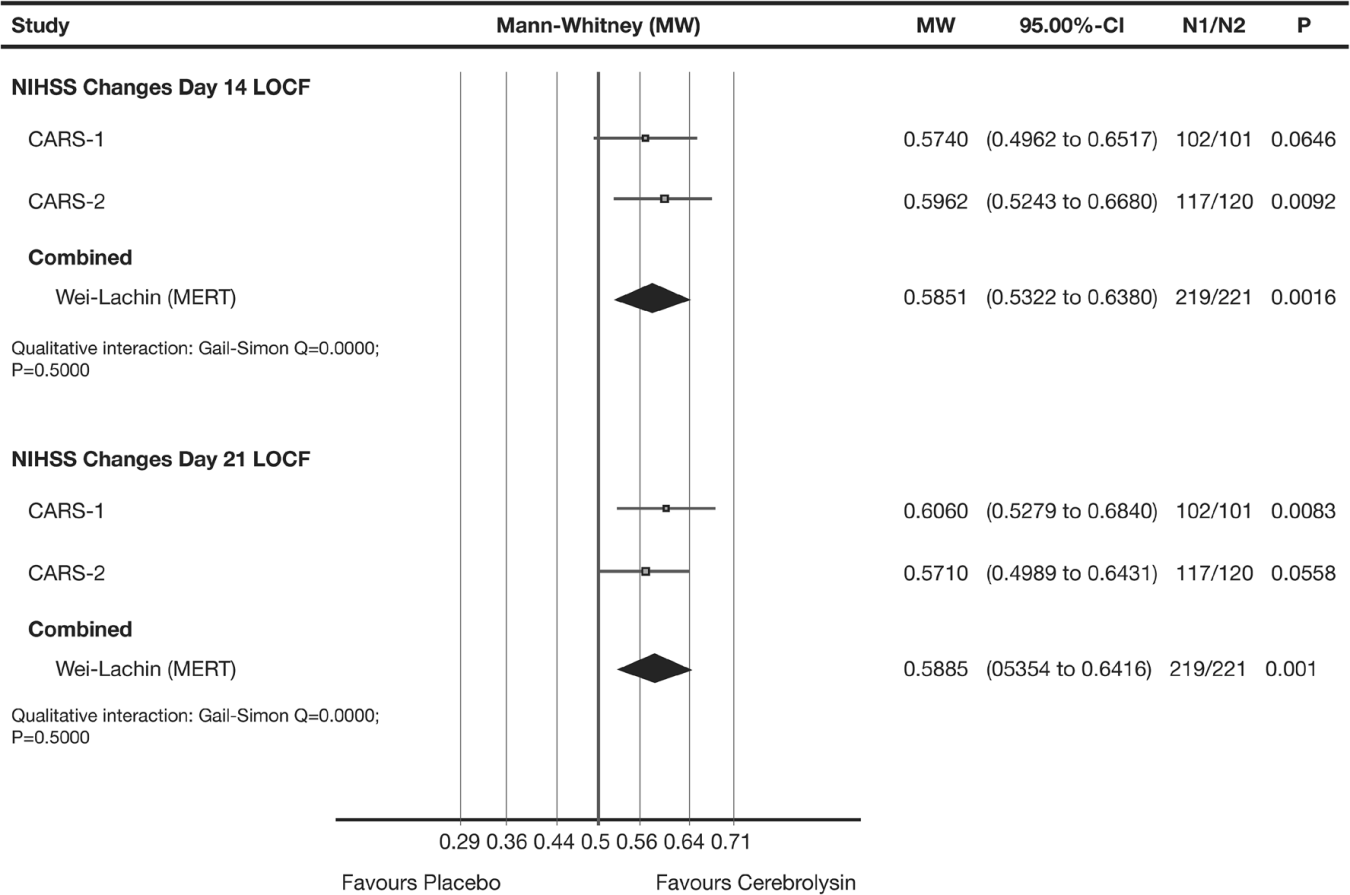
Meta-analysis of early NIHSS changes from baseline of CARS-1 and CARS-2. Comparison of Cerebrolysin (30 ml/day) versus placebo at day 14 (*upper panel*) and day 21 (*lower panel*) in mITT population; LOCF. Wei-Lachin pooling procedure, maximin-efficient robust test (MERT), effect size: Mann-Whitney (MW)

Early benefit was evaluated by the changes from baseline of the NIHSS [18] at day 14 and day 21. The evaluations for early benefit by means of the NIHSS showed throughout statistical significance (day 14: *P* = 0.0016, day 21: *P* = 0.0010, Wei-Lachin pooling procedure [MERT], Fig. 3).

Clinical relevance was analysed applying the original NINDS definition [35] (NIHSS change of at least 4 points or resolution of symptoms). The result of the corresponding meta-analysis, based on the odds ratios (OR) at day 21, was statistically significant in favour of Cerebrolysin (OR = 1.805; 95% CI 1.19 to 2.73; *P* = 0.0053, *I* ^2^ = 0%). The rate difference regarding clinically relevant NIHSS changes was 17.3% in CARS-1 and 11.0% in CARS-2, both favouring Cerebrolysin. The combined number-needed-to-treat (NNT) for clinically relevant changes in early NIHSS was 7.1 (95% CI: 4 to 22).

### Safety and tolerability

A total of 96.2% of the patients treated in CARS-1 and CARS-2 received 21 infusions (Cerebrolysin, 96.9%; placebo, 95.5%). Of the patients treated with Cerebrolysin, 46.0% reported at least one adverse event (AE) compared with 41.5% of the patients in the placebo group. Most of the AEs were rated mild in severity (Cerebrolysin, 65.9%; placebo, 66.8%). Most frequent treatment-emergent adverse events (TEAEs) reported in at least 5% of the patients in any group were urinary tract infection (5.8% in the Cerebrolysin group and 7.6% in the placebo group) and depression (8.5% in the Cerebrolysin group and 6.3% in the placebo group). Eleven patients (4.9%) in each group suffered from serious adverse events (SAEs), but none of the SAEs were rated as related to study medication. In the Cerebrolysin group the outcome of all SAEs was described as resolved or resolved with sequelae except for two patients (0.9%) who died due to pneumonia and ischemic stroke. In the placebo group, four patients (1.8%) died due to sepsis with acute renal failure and coma, sepsis with multi-organ failure, intestinal ischemia and subdural plus intracerebral hematoma.

Vital signs were similar between the treatment groups, and these factors did not show clinically relevant changes during the course of the study. The laboratory values classified by the investigators as clinically relevant did not exhibit any significant differences between the treatment groups, and no trends toward specific pathological laboratory findings were detected. Overall, the safety outcome reflected the expected safety and tolerability profile of patients after acute ischemic stroke.

### Sensitivity analyses

#### Observed Case analysis

Since missing values and dropouts represent a risk of bias, an observed case (OC) analysis was performed as pre-planned sensitivity analysis. The OC result of the primary efficacy criterion ARAT is well supporting the primary LOCF analysis (MW_OC_ 0.62 with P_OC_ < 0.0001 as compared to MW_LOCF_ 0.62 with P_LOCF_ < 0.0001). The consistency of the results is expected since the OC population was 97.1% as compared to the full mITT population, minimizing the corresponding risk of bias.

The same applies to the OC result for the pre-defined target subset (ARAT >0) at day 90, which is again well comparable to the LOCF analysis (MW_OC_ 0.61 with P_OC_ < 0.0023 as compared to MW_LOCF_ 0.61 with P_LOCF_ < 0.0015).

#### ‘Classic’ synthesis

A second line ‘classic’ fixed and random effects analysis has been performed in addition to the robust first line approach of stochastic superiority (MERT). All in all, the effect sizes for the ARAT changes from baseline at day 90 based on fixed effect and random effects compare very well to the primary Wei-Lachin pooling procedure (MERT) with MW_fixed_ 0.61 and MW_random_ 0.62 as compared to MW_MERT_ 0.62 (Fig. 2, upper panel). While the fixed effect is statistically significant (*P*_fixed_ < 0.0001, 95% CI 0.56 to 0.67) and, thus, again compares well to the result of the Wei-Lachin pooling procedure (MERT) with *P*_MERT_ < 0.0001, the random effects result fails to do so (*P*_random_ 0.1791, 95% CI 0.44 to 0.80). This deviation should be interpreted with certain care due to the inappropriateness of the random approach to estimate between-study variance properly with only two included studies [29–31].

While the test for qualitative interaction suggests that there is no indication for opposite effects (Gail-Simon test *P* = 0.5), the test for quantitative interaction indicates serious heterogeneity in the full mITT population with respect to the final ARAT results at day 90 (*I* ^2^ = 0.90). This heterogeneity may be due to the markedly milder stroke severity in CARS-2, allowing good recovery also in the placebo group. The pre-planned analysis of the target subset (exclusion of ARAT zero scores at baseline) markedly reduces the impact of the baseline heterogeneity of the two studies (MW_fixed_ 0.60 and MW_random_ 0.61, as compared to MW_MERT_ 0.61, Fig. 2, lower panel; all *P* < 0.05 two-sided), and quantitative heterogeneity at day 90 is reduced below 50% in the target subset (*I* ^2^ = 0.46). More details with respect to the structural heterogeneity of the two studies and further sensitivity analyses are provided in the “Discussion” section.

#### Adjustment for gender, age and ARAT baseline

Stratified analyses for gender, for quartiles of baseline ARAT and for age were defined as further sensitivity analyses for the primary efficacy criterion ARAT. Adjustment for these confounders was performed on individual study level for CARS-1 and CARS-2. The meta-analyses of the adjusted results showed that there was no indication for a confounding impact, all adjusted results were well comparable to the unadjusted result (gender: MW_adj_ 0.62, age: MW_adj_, 0.63, ARAT baseline: MW_adj_ 0.62, as compared to MW_unadj_ 0.62, all *P* < 0.0001). The same applies to the corresponding OC analyses.

## Discussion

This meta-analysis combines the results of two identical studies with Cerebrolysin in early rehabilitation patients after stroke. Both studies were placebo-controlled, double-blind, and parallel-group trials with a treatment period of 21 days. Patients were randomly assigned to either Cerebrolysin or placebo, and treatment arms were equally balanced with regard to demographic characteristics. All analyses have been performed using individual patient data (IPD). Cerebrolysin treatment resulted in a statistically significant benefit in the pre-planned nonparametric meta-analysis of the primary efficacy criterion ARAT (Wei-Lachin pooling procedure [MERT]), in the full mITT population as well as in the pre-planned target subset of patients with ARAT baseline score > 0. The rate of premature discontinuations was below 5% in both studies (CARS-1, 3.8%; CARS-2, 4.2%), adding to the validity of each of the studies individually and the resulting meta-analysis.

While treatment groups were well comparable at baseline within each single study, and early effect sizes for early improvements were highly consistent, the final results at day 90 showed considerable heterogeneity of effect sizes due to the marked final ceiling effects in the placebo group of CARS-2. The ceiling effects may well be explained by the substantially milder baseline levels allowing good final outcome also in the placebo group of CARS-2. In order to better understand the structural differences between the two studies and also to learn for future study designs and treatment concepts, the baseline situation shall be discussed in more detail, using the highly deviating results of the two placebo groups (final placebo ARAT median of 53.0 in CARS-2 as compared to 27.0 in CARS-1).

In-depth comparison of the placebo distributions of the two studies by means of the cumulative distribution function revealed that CARS-2 has substantially milder baseline levels as compared to CARS-1 throughout the whole ARAT distribution (strong right shift of the CARS-2 curve). This is also expressed by the associated ARAT baseline medians: 33.0 (‘mildʼ) [36] in CARS-2 as compared to only 2.0 (‘severeʼ) [36] in CARS-1.

Additional baseline characteristics underline the heterogeneity of the two placebo populations: with ARAT baseline zero scores (worst score) in 20 vs 49% of the subjects and an NIHSS baseline level ≤7 in 70% (CARS-2) vs 30% (CARS-1) of the subjects, baseline differences of the two studies are in fact substantial and well explain the good placebo results in CARS-2.

This is also made evident by the motor subscore of the NIHSS: an initial score of 1, indicating only minor impairment, was found in 16.8% of placebo patients in CARS-1 but in 58.3% of placebo patients in CARS-2 (with a mean baseline NIHSS motor score for paretic arm of 2.6 in CARS-1 as compared to 1.7 in CARS-2). It is important to note that NIHSS or single NIHSS items were not an inclusion or exclusion criterion; the inclusion criterion regarding motor arm deficits was based on the primary efficacy criterion ARAT.

Together with the observed ceiling effects in CARS-2 (see boxplots in Fig. 1, lower panel), the different baseline situation well explains the heterogeneity of the effect sizes for the comparison of Cerebrolysin vs placebo. Where patients are already in a ‘mildʼ severity status at baseline, also placebo may reach after 90 days the ceiling of the outcome scales.

A major source of baseline heterogeneity could be eliminated by discarding the ‘zero’-ARAT scores (worst possible ARAT score). The ‘zero-problem’ and its corresponding floor effect were discussed in the past by various researchers [37–39]. As expected, the heterogeneity of the studies could substantially be reduced in the target subset (*I* ^2^ reduction from >90 to <50%), and the meta-analysis of the two studies became statistically significant also in the random effects model (*P* = 0.0288, two-sided).

Nijland et al. [39] defined floor and ceiling benchmarks for the ARAT score as <3 points (floor) and >54 points (ceiling). Sensitivity analysis applying these benchmarks to the baseline ARAT scores for defining a more homogeneous subset of the overall population showed statistical significance in all analyses including the random effects model (MW 0.61, 95% CI 0.53 to 0.68, *P* = 0.0068, Wei-Lachin pooling procedure [MERT]; MW 0.59, 95% CI 0.52 to 0.66, *P* = 0.0110, fixed effects; MW 0.59, 95% CI 0.52 to 0.66, random effects, *I* ^2^ = 0.0). Thus, excluding the outlying baseline patients according to Nijland [39], heterogeneity between the two studies was reduced even in a more dramatic way than with sole exclusion of the ARAT baseline zero values, leading to a marked reduction of *I* ^2^ from >90 to 0%. It may be useful to apply these benchmarks also in future studies in order to control heterogeneity and to obtain more precise results. More restrictive benchmarks (especially at the upper end of the ARAT scale) could not be evaluated due to the low number of patients within the potential windows of interest.

Due to the final ceiling effects in the placebo group of CARS-2 (day 90), the analyses of earlier points in time were of special interest regarding impact on heterogeneity: placebo effects might not yet have reached the ceiling of good recovery—thus, leaving room for detection of additional beneficial treatment effects and potentially converging the results of the two studies.

As noted previously, the NIHSS is most sensitive for such earlier points in time [19]. The analyses for early benefit by means of the NIHSS showed statistical significance in all analyses including the ‘classic’ fixed effect and random effects models (day 14: *P* = 0.0016, Wei-Lachin pooling procedure [MERT]; *P* = 0.0014 fixed effects, *P* = 0.0014 random effects, *I* ^2^ = 0.0; day 21: *P* = 0.001, Wei-Lachin pooling procedure [MERT]; *P* = 0.0013 fixed effects, *P* = 0.0013 random effects, *I* ^2^ = 0.0). Thus, between-study heterogeneity was completely resolved. This confirms the findings of Kerr et al. (2012) [19], highlighting the importance of early NIHSS for future research in stroke due to greater sensitivity and better control of background noise.

A supportive analysis on final global disability was performed by means of the modified Rankin scale (mRS) since this is the most commonly used functional measure in stroke trials. The nonparametric analysis of the mRS showed statistical significance at the final visit (day 90) with combined MW 0.61 and *P* < 0.0001 in the mITT population (Wei-Lachin pooling procedure [MERT]). The analysis of the Nijland subset (see above) resulted in an identical effect size (MW 0.61; *P* = 0.0031). As expected from the markedly milder initial stroke severity in CARS-2, substantial heterogeneity of final mRS effect sizes was found at the end of the two trials, even in the more homogeneous Nijland subset (excluding ARAT baseline outliers): while CARS-1 showed strong treatment effects with MW 0.70, CARS-2 indicated only weak effects with MW 0.52. The difference is well explainable by the cited ceiling effects in CARS-2, preventing sufficient assay sensitivity for final mRS. Further trials with higher initial stroke severity are recommended to obtain better insight into the size of treatment effects with respect to long-term functional outcome.

## Summary and conclusions

This meta-analysis provides evidence that Cerebrolysin has a beneficial effect on motor function recovery in early rehabilitation patients after stroke. The primary meta-analytic result (ARAT at day 90) was statistically significant in the full mITT population (*P* < 0.0001) as well as in the pre-planned target subset (*P* = 0.0015). While there was some study heterogeneity, well explainable by the different initial stroke severity with substantially milder cases in CARS-2, sensitivity analyses for patients within the ARAT floor-ceiling benchmarks according to Nijland [39] reduced heterogeneity to 0% (*I* ^2^), showing consistent statistical significance in all analytical models. Also, the analysis of early benefit at day 14 and day 21 by means of the NIH stroke scale (NIHSS), which is regarded as most sensitive to early improvements [19], showed statistical significance throughout all analytical approaches. The number-needed-to-treat (NNT) for clinically relevant changes in early NIHSS was 7.1 (95% CI 4 to 22), with a 17.3% rate difference in CARS-1 and a 11.0% rate difference in CARS-2.

The safety characteristics of Cerebrolysin were comparable to placebo, thus suggesting a favourable benefit-risk ratio.

Cerebrolysin as a therapeutic agent should be considered by clinicians seeking early recovery treatment options after stroke. Due to the exploratory character of the two trials, the results should be confirmed by a broader ensemble of trials with sufficient initial stroke severity. Furthermore, introduction of proper inclusion criteria of the ARAT score, avoiding floor and ceiling effects, may be considered for future efficacy studies. The positive and very homogeneous results on early benefit (NIHSS) may encourage future early-phase research on therapeutic interventions after acute ischemic stroke.
